# Rare Case of Multiple Intradural Extramedullary Spinal Schwannomas With Intramedullary Extension

**DOI:** 10.7759/cureus.13228

**Published:** 2021-02-08

**Authors:** Gasim Ahmed, Usman Sheikh, Timothy Dawson, Hemant Sonwalker

**Affiliations:** 1 Radiology, The Christie Hospital NHS Foundation Trust, Manchester, GBR; 2 Radiology, Lancashire Teaching Hospitals NHS Foundation Trust, Preston, GBR; 3 Pathology, Lancashire Teaching Hospitals NHS Foundation Trust, Preston, GBR

**Keywords:** intradural, extramedullary, spinal, schwannoma, intramedullary extension, neuroimaging

## Abstract

Spinal schwannomas are benign WHO grade I nerve sheath tumors that account for nearly 30% of all spinal neoplasm. Typically, these lesions are intradural extramedullary in location and are composed entirely of well-differentiated eosinophilic Schwann cells. Intramedullary schwannomas, however, are extremely rare due to the lack of Schwan cells in the normal spinal cord and represent 1% of all the spinal schwannoma population. The presence of such an intramedullary component makes diagnosis challenging as imaging features may resemble other intramedullary neoplastic entities.

Here, we describe a case of a 56-year-old male patient who presented with an 18-month history of intermittent right-sided mid-thoracic pain secondary to multiple intradural extramedullary spinal schwannoma with intramedullary extensions. We also review the literature pertaining to the condition.

## Introduction

Schwannomas are benign, firm, encapsulated, Schwann cell-derived nerve sheath tumors that commonly develop outside the central nervous system (CNS), usually involving peripheral nerves and subcutaneous tissue. When these classically intradural extramedullary neoplasms arise within the CNS, they account for nearly 25% to 30% of spinal nerve root tumors. The vast majority of schwannomas (95%) are solitary and sporadic, whereas multiple schwannomas are associated with neurofibromatosis 2 (NF2) and schwannomatosis. Although spinal schwannomas may occur at any age, they classically express a peak incidence in the fifth to sixth decades of life and do not display gender predilection [1].

Intramedullary extension of spinal schwannoma on the other hand is very rarely reported in the literature. Unlike its classical extramedullary counterpart, when such intramedullary extension is present, these neoplasms express a male-to-female ratio of 3:1 and preferentially present in the fourth decade of life. The possession of an intramedullary component makes differentiating such spinal schwannomas from other intramedullary spinal neoplasms a radiological challenge, and thus it is of great importance to review the imaging characteristics and presentation of such a rare entity [1,2].

## Case presentation

A 56-year-old Caucasian male with no significant past medical history presented with an 18-month history of intermittent sharp shooting-type right-sided chest pain. The pain was aggravated by arm movement and radiated to the anteromedial aspect of the right pectoral region. The patient denied any other symptomatology. Physical examination was unremarkable. In particular, no upper or lower limb motor or sensory deficit was detected. Routine laboratory evaluation (full blood count C-reactive protein, erythrocyte sedimentation rate) and a chest X-ray were normal.

**Figure 1:**
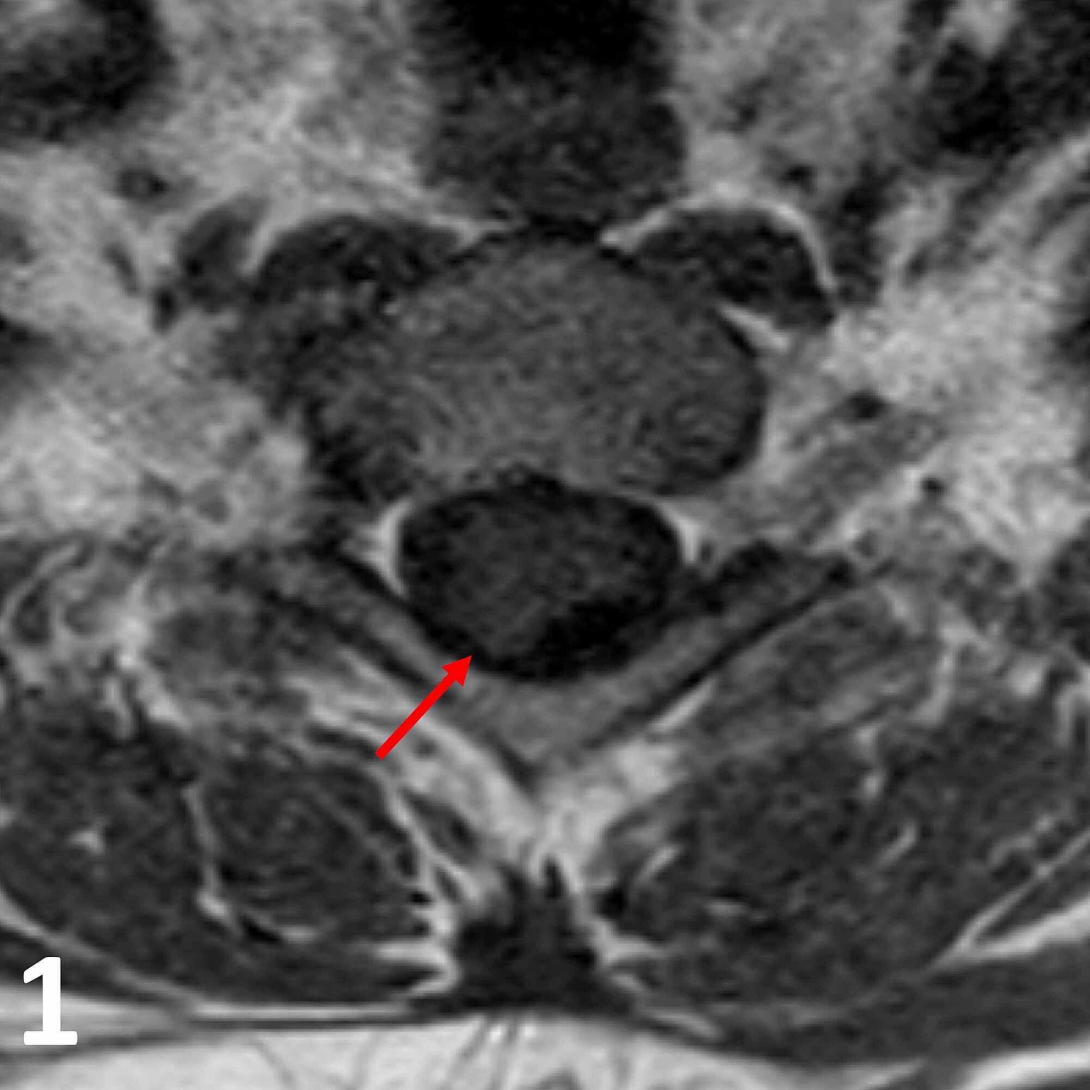
Axial T1 pre-contrast MRI image at the T1 vertebral level. Note the T1 isointense intradural lesion (red arrow) in the right posterolateral aspect of the spinal cord.

**Figure 2:**
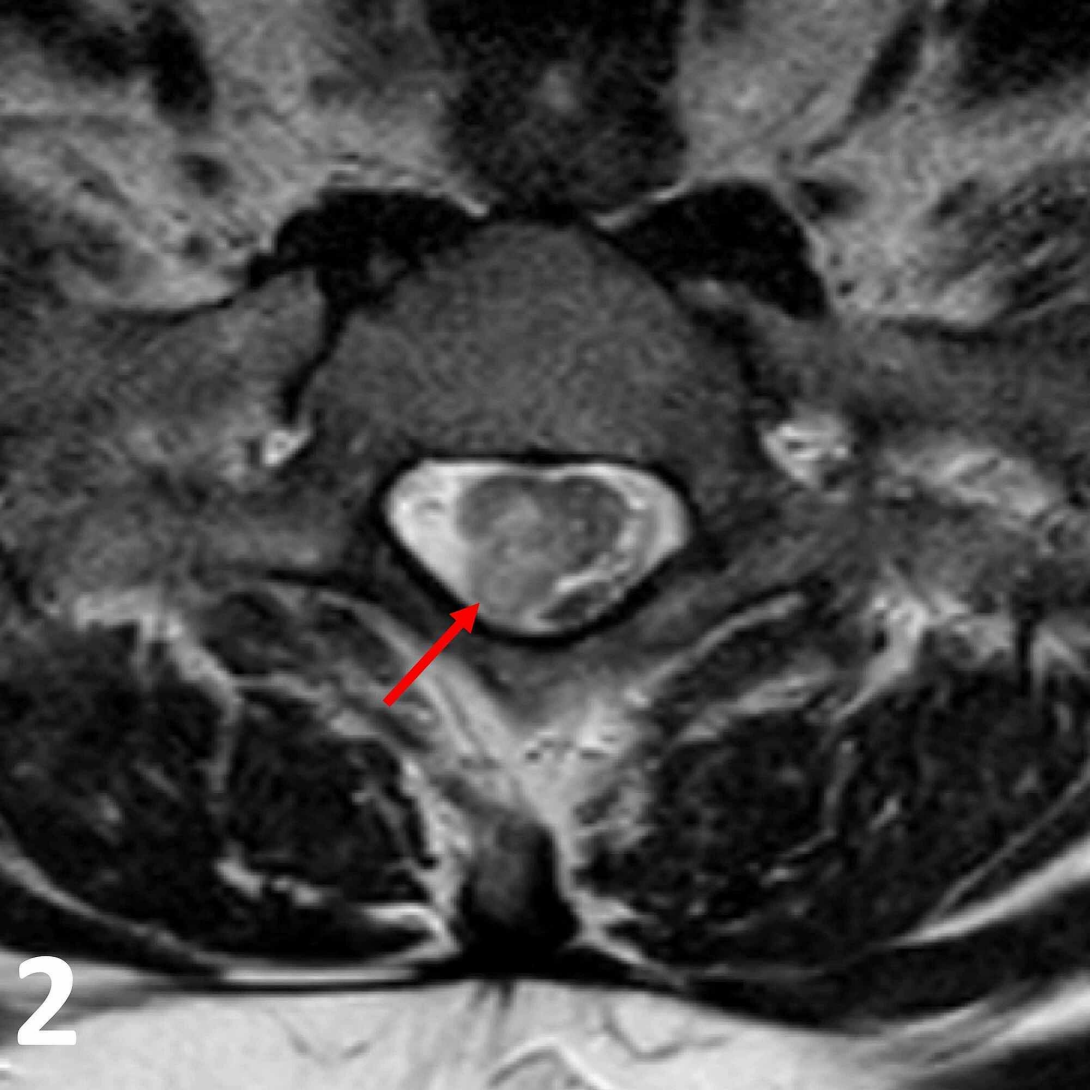
Axial T2 MRI image at the T1 vertebral level. Note the T2 heterogeneously hyperintense intradural lesion (red arrow) in the right posterolateral aspect of the spinal cord. CSF (T2 hyperintense signal) can be seen surrounding the spinal cord, signifying no spinal cord compression. CSF, cerebrospinal fluid

**Figure 3:**
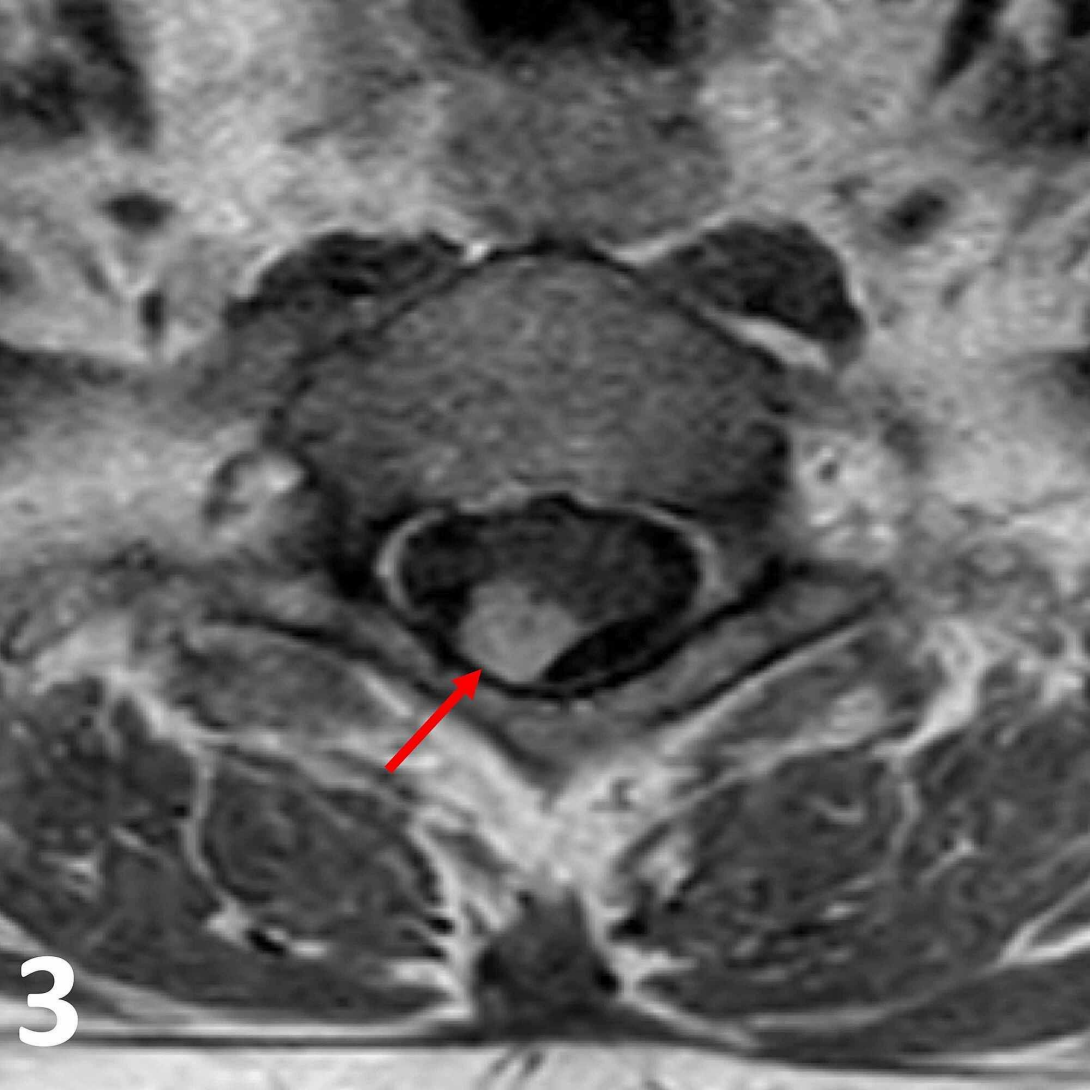
Axial T1 post-gadolinium MRI image at the T1 vertebral level. Note the homogenously enhancing intradural lesion (red arrow) in the right posterolateral aspect of the spinal cord. The lesion exhibits both intramedullary and extramedullary components, which are rarely reported in schwannomas.

A magnetic resonance imaging (MRI) scan of the brain was normal. A spinal MRI scan was carried out for further assessment. At the T1 vertebral level, a 9-mm T1 isointense, T2 heterogeneously hyperintense, homogenously enhancing intradural extramedullary mass with an intramedullary component was detected. The lesion involved the right posterolateral aspect of the spinal cord and exhibited a posterior exophytic component. The mass displayed no broad-based dural component, cystic or hemorrhagic features, flow voids, calcifications, or spinal cord expansion (Figures 1-3).

**Figure 4:**
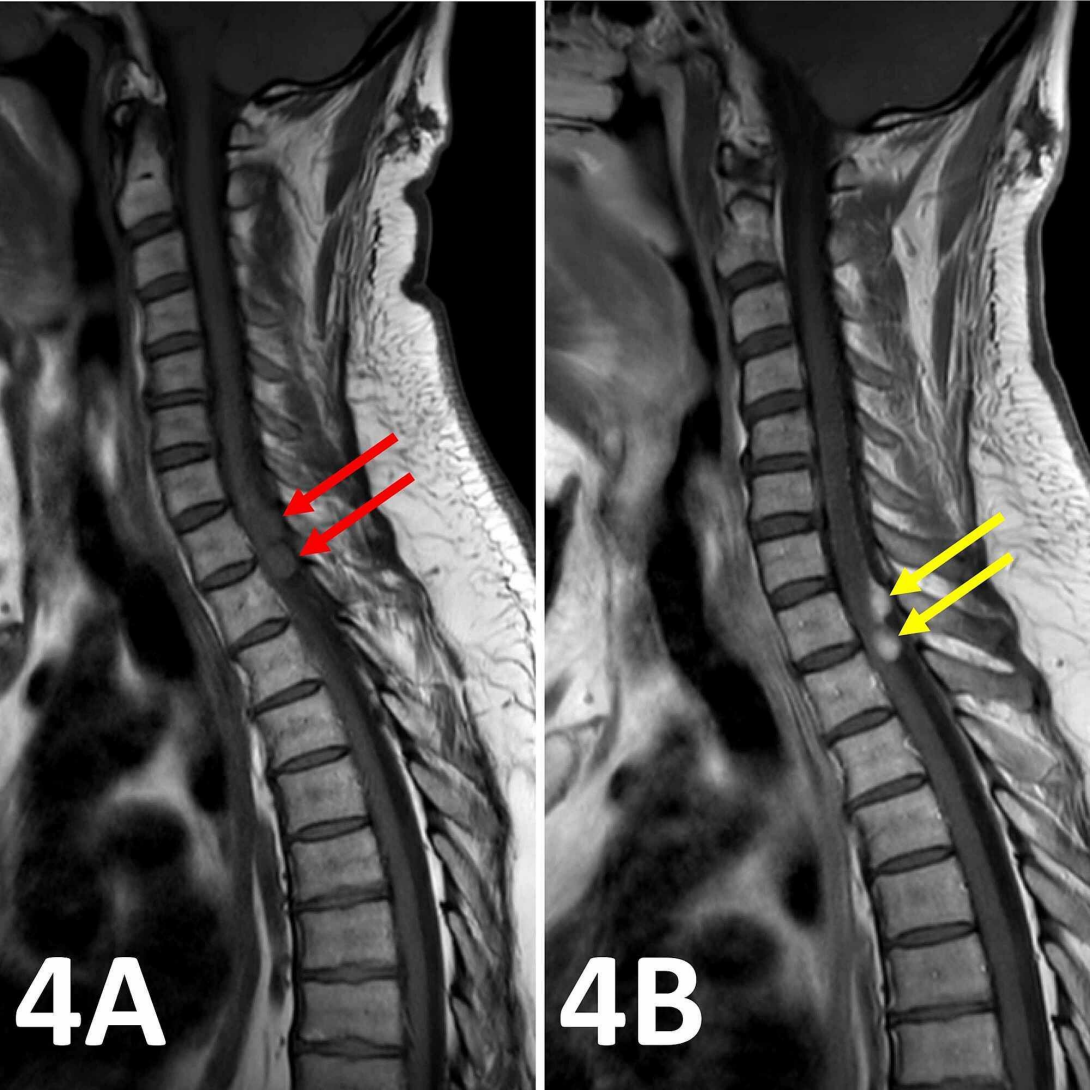
Sagittal T1 pre- and post-contrast spinal MRI images. (A) Sagittal T1 pre-contrast spinal MRI image showing two isointense intradural lesions at the T1/T2 vertebral level (red arrows). (B) Post-gadolinium administration, the two lesions enhance homogenously highlighting their intramedullary and extradural elements (yellow arrows).

**Figure 5:**
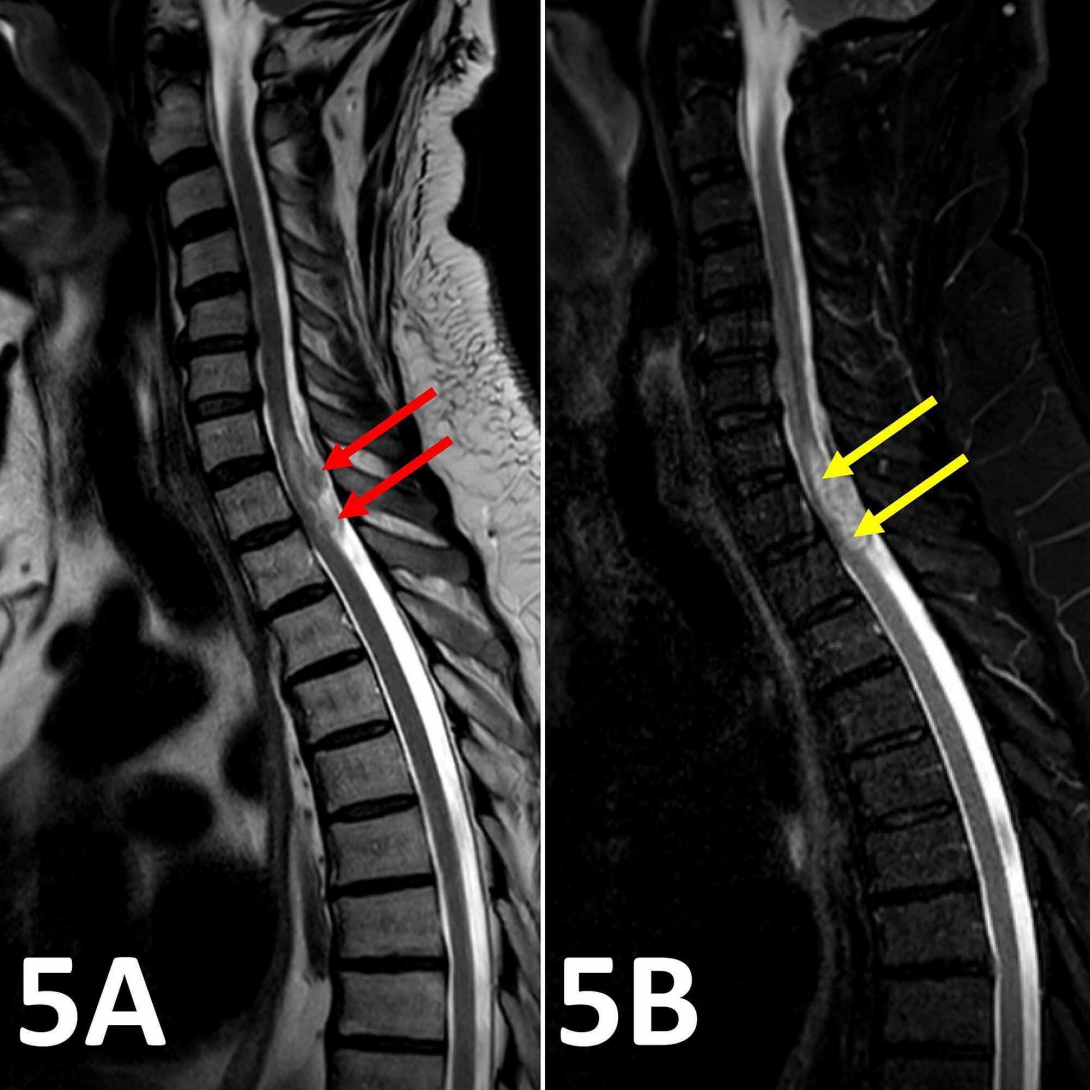
Sagittal T2 and T2 fat-suppressed spinal MRI images. (A) Sagittal T2 spinal MRI image showing the heterogeneously hyperintense characteristics of the two intradural lesions at the T1/T2 vertebral level (red arrows). (B) The T2 fat-suppressed sagittal MRI spine image at the same level reveals no suppression of the high T2 central cord signal signifying mild cord edema (yellow arrows).

**Figure 6:**
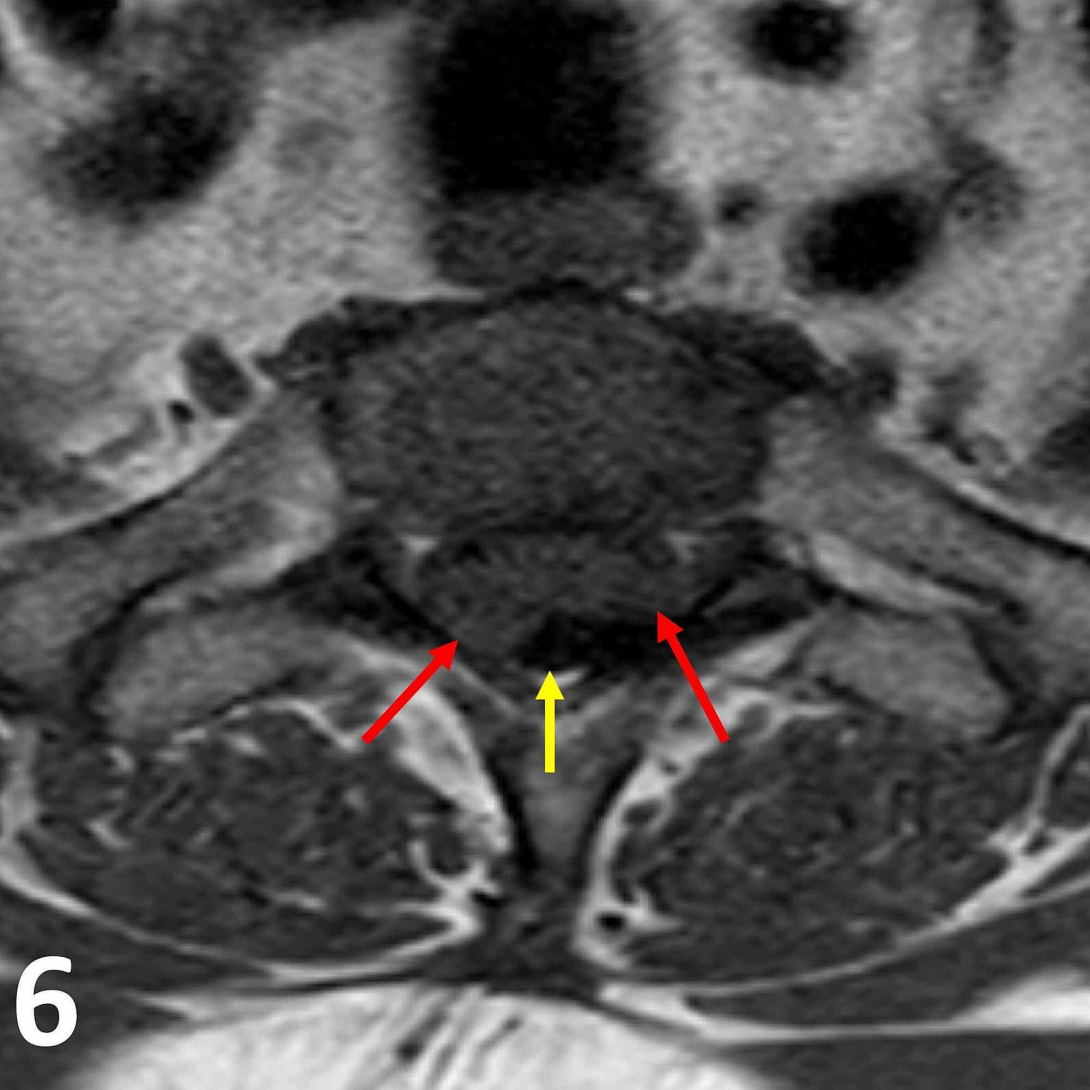
Axial T1 pre-contrast MRI image at the T1/T2 vertebral level. Note the T1 isointense intradural lesions (red arrows) projecting posterolaterally within the thecal sac (yellow arrow).

**Figure 7:**
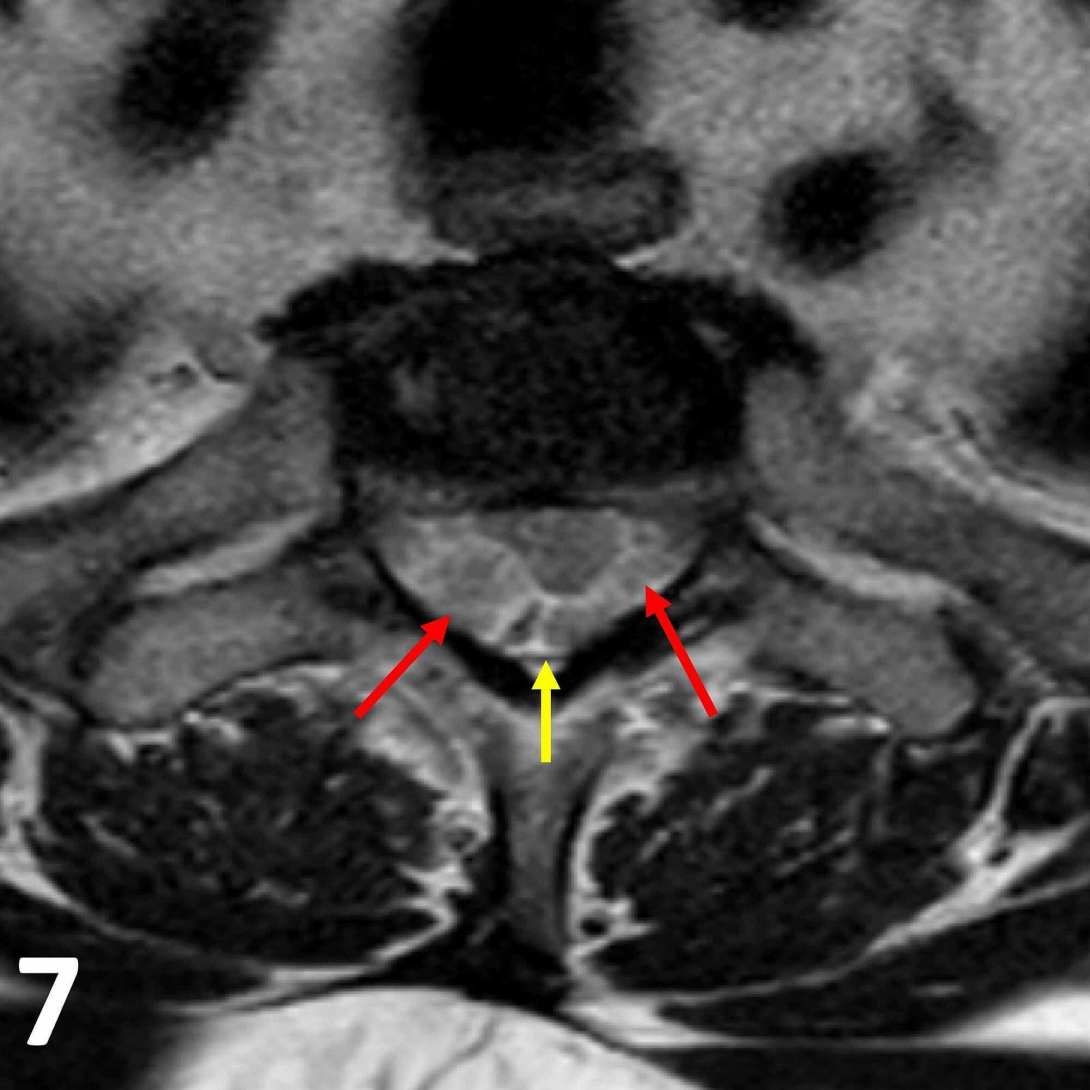
Axial T2 MRI image at the T1/T2 vertebral level. Note the T2 heterogeneously hyperintense intradural lesions (red arrows) within the thecal sac (yellow arrow). The two lesions, particularly the larger right-sided one, are indenting the spinal cord without causing spinal cord compression.

**Figure 8:**
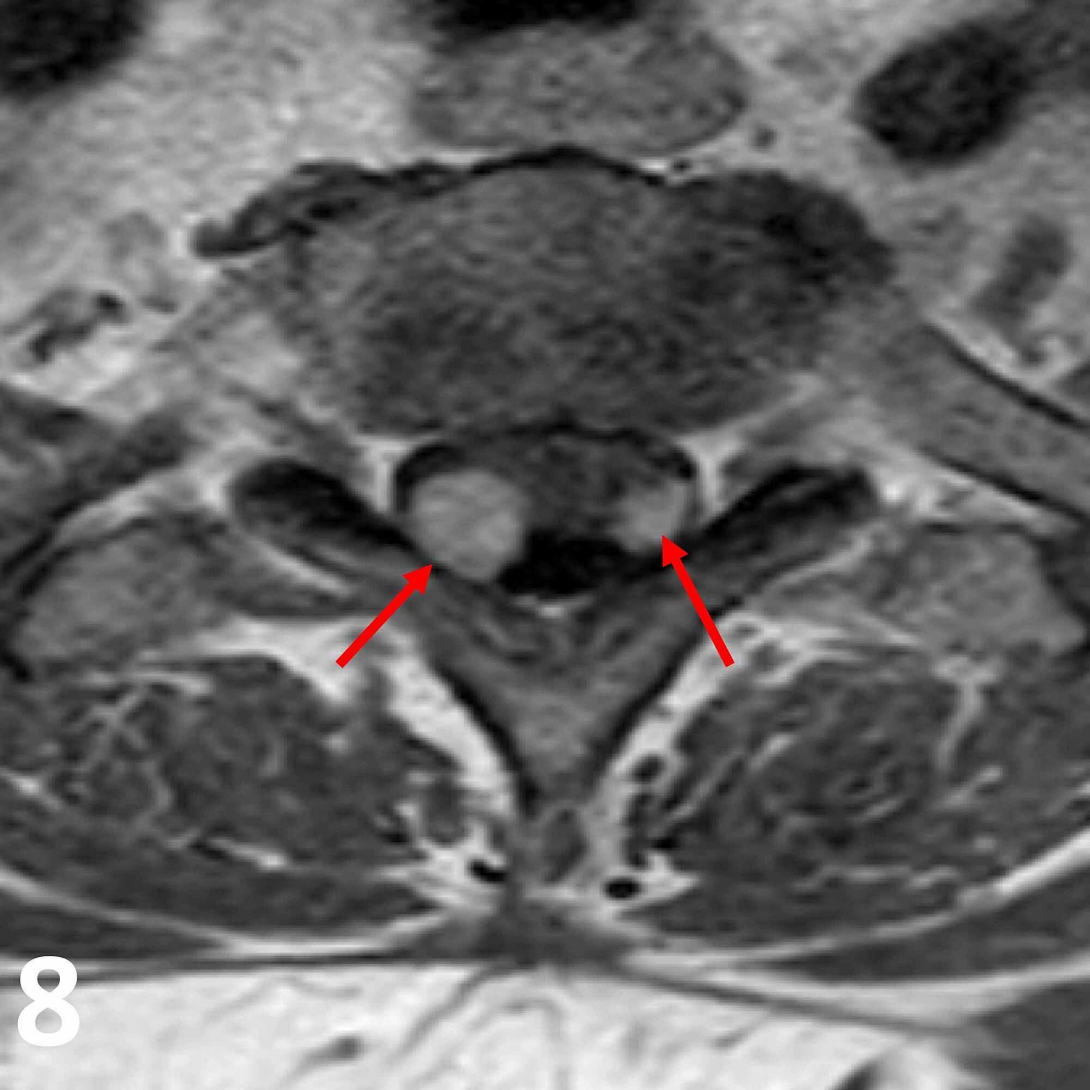
Axial T1 post-contrast MRI image at the T1/T2 vertebral level. Note the homogenously enhancing intradural lesions (red arrows) in the right posterolateral aspect of the spinal cord. Both lesions exhibit intramedullary and extramedullary components, which are unusual and rare for schwannomas.

At the T1/T2 vertebral level, two additional 9- and 5-mm intradural lesions with both an intra- and extramedullary components were noted. These lesions demonstrated similar MRI characteristics to the lesion at the T1 level. The smaller left-sided lesion projected along the course of nerve roots in the posterolateral aspect of the cord, whereas the larger right-sided lesion indented the cord. There was no evidence of cord compression (Figures 4-8).

As there were no red flag features (focal neurological deficit or radiological cord compression), a conservative management approach was adopted and future annual MRI follow-up was planned. Two years later, the patient presented with significant neuropathic chest pain refractory to analgesics. A spinal MRI scan showed a 2-mm increase in the size of the spinal lesions with associated spinal cord edema. As a consequence, laminectomy and tumor excision was carried out. Intra-operatively, multiple separate intradural lesions with intramedullary extension were identified corresponding to the filing detected on MRI. The vast majority of lesions were resected, with only a small remnant left to avoid iatrogenic spinal cord injury.

**Figure 9:**
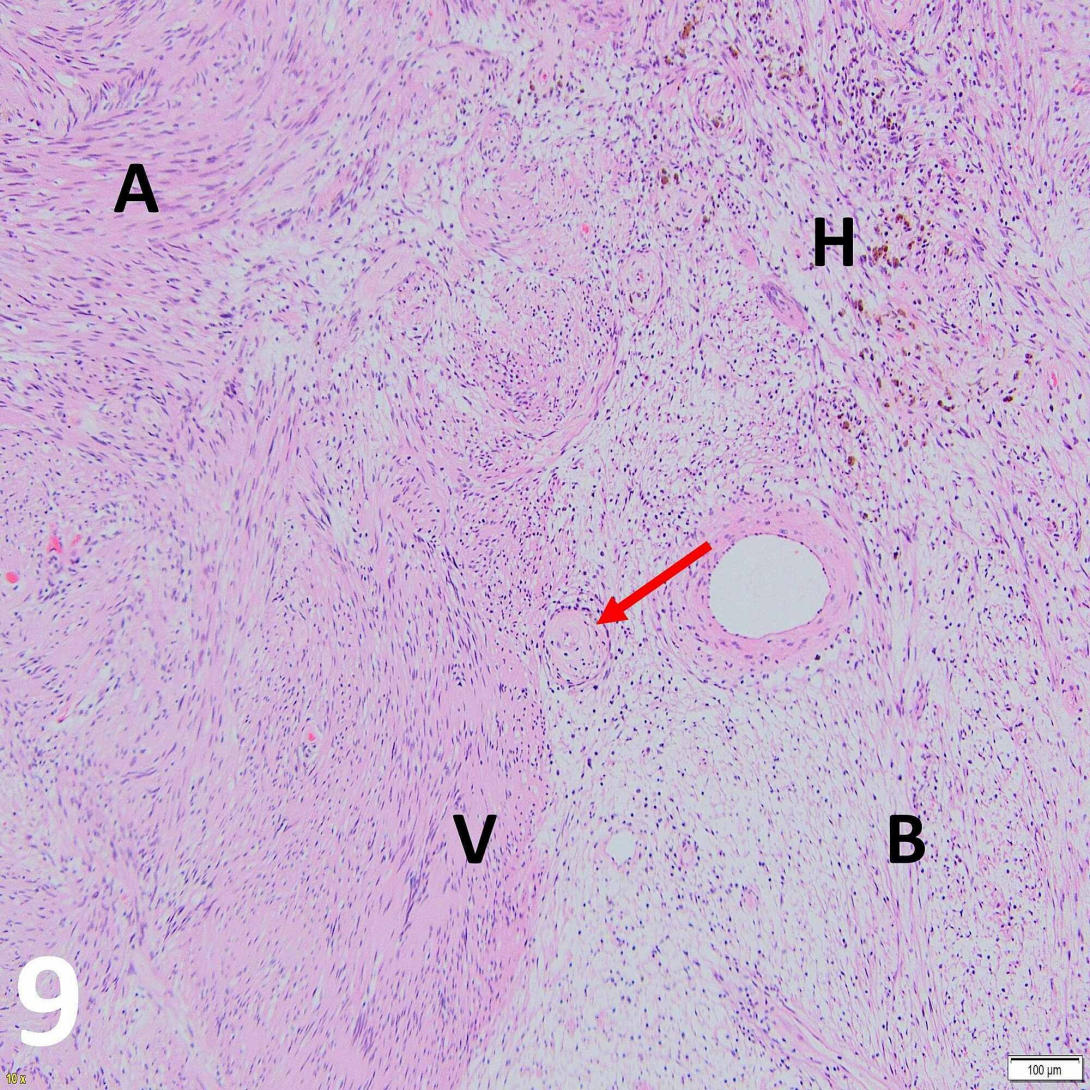
Histopathology H&E stained imaged at x10 objective magnification demonstrating the main features of schwannomas. (A) A dense spindled cell Antoni A area. (B) A pauci-cellular, microcystic Antoni B area. The red arrow indicates vascular mural hyalinization often seen in schwannomas and (H) the accompanying hemosiderin deposits resulting from micro-hemorrhages due to vessel wall fragility. (V) An area of nuclear palisading with intervening amorphous eosinophilia characteristic of a Verocay body.

**Figure 10:**
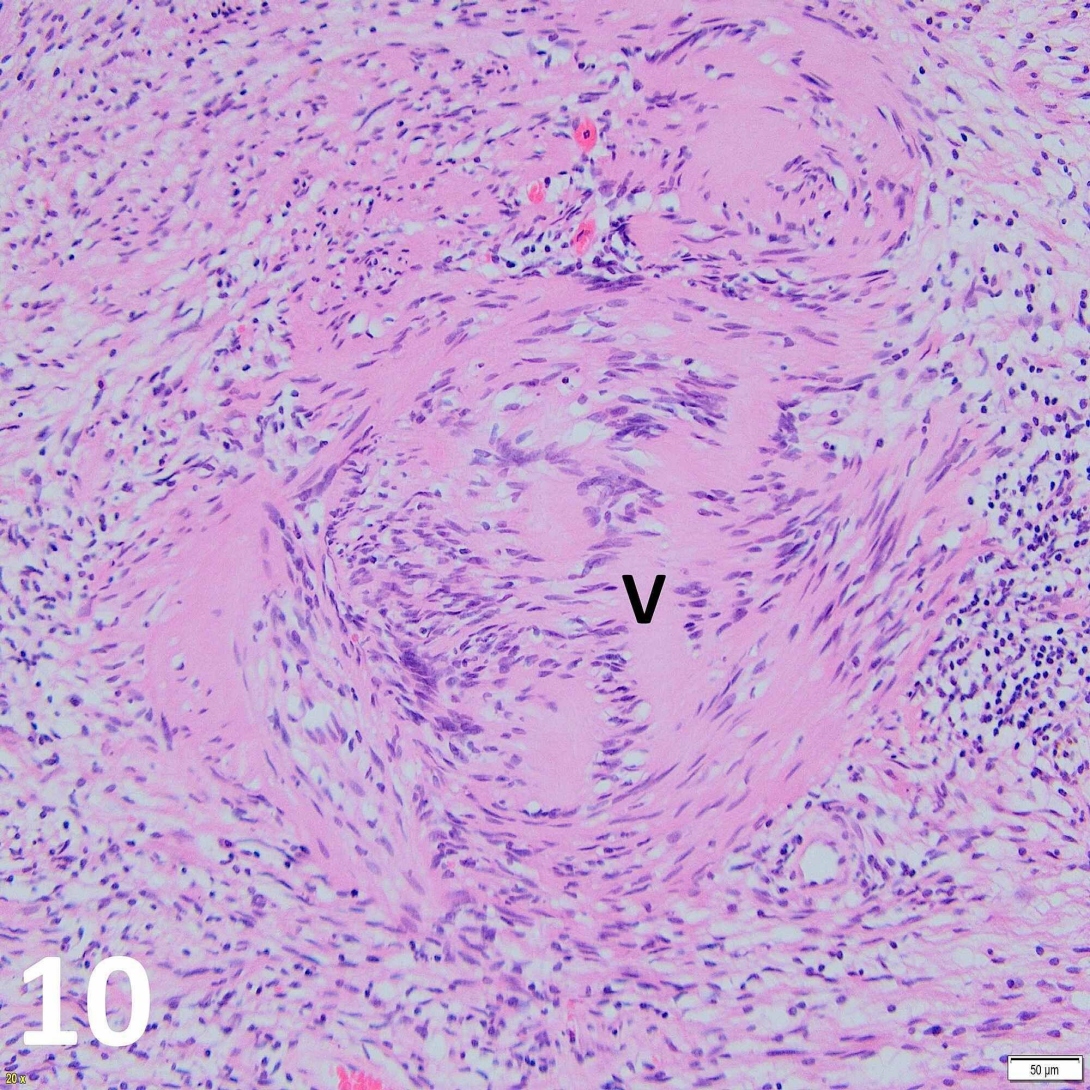
Histopathology H&E stained imaged at x20 objective magnification. Note the pathognomic Verocay body (V) believed to be an aberrant recapitulation of Pacinian corpuscle morphology.

**Figure 11:**
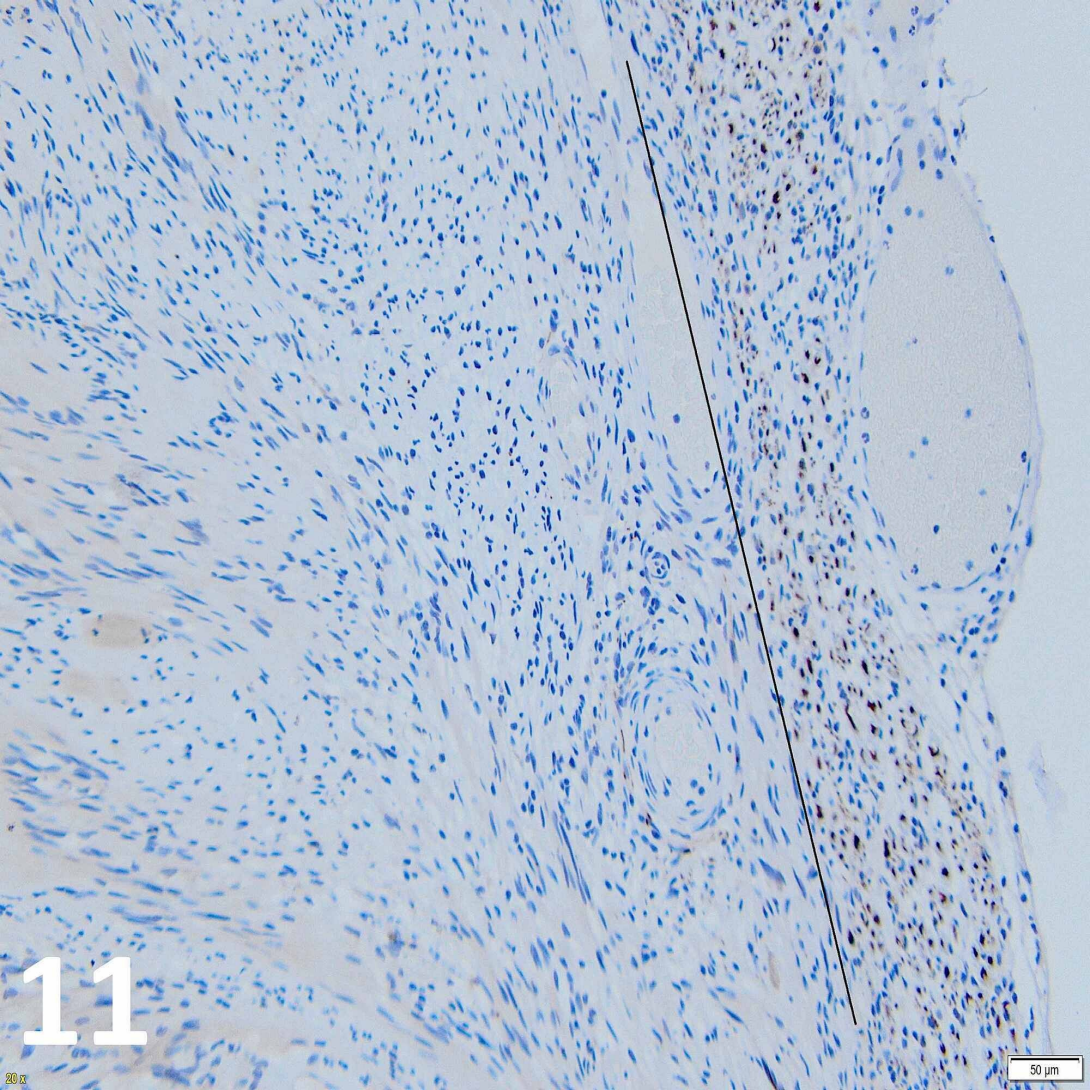
Neurofilament protein immunostaining at x20 objective magnification. Neurofilament protein immunostaining demonstrated by DAB brown polymer deposits in axons (to the right of the line) showing how the native nerve root is pushed to the lesion periphery by tumor growth.

Histopathological evaluation of the excised tumor showed biphasic architecture with areas of densely compacted spindle cells with pronounced nuclear palisading, whereas other areas demonstrated less cellularly dense architecture. No mitotic activity was seen. Features were consistent with Antoni A and B growth patterns of schwannoma (Figures 9-11).

**Figure 12:**
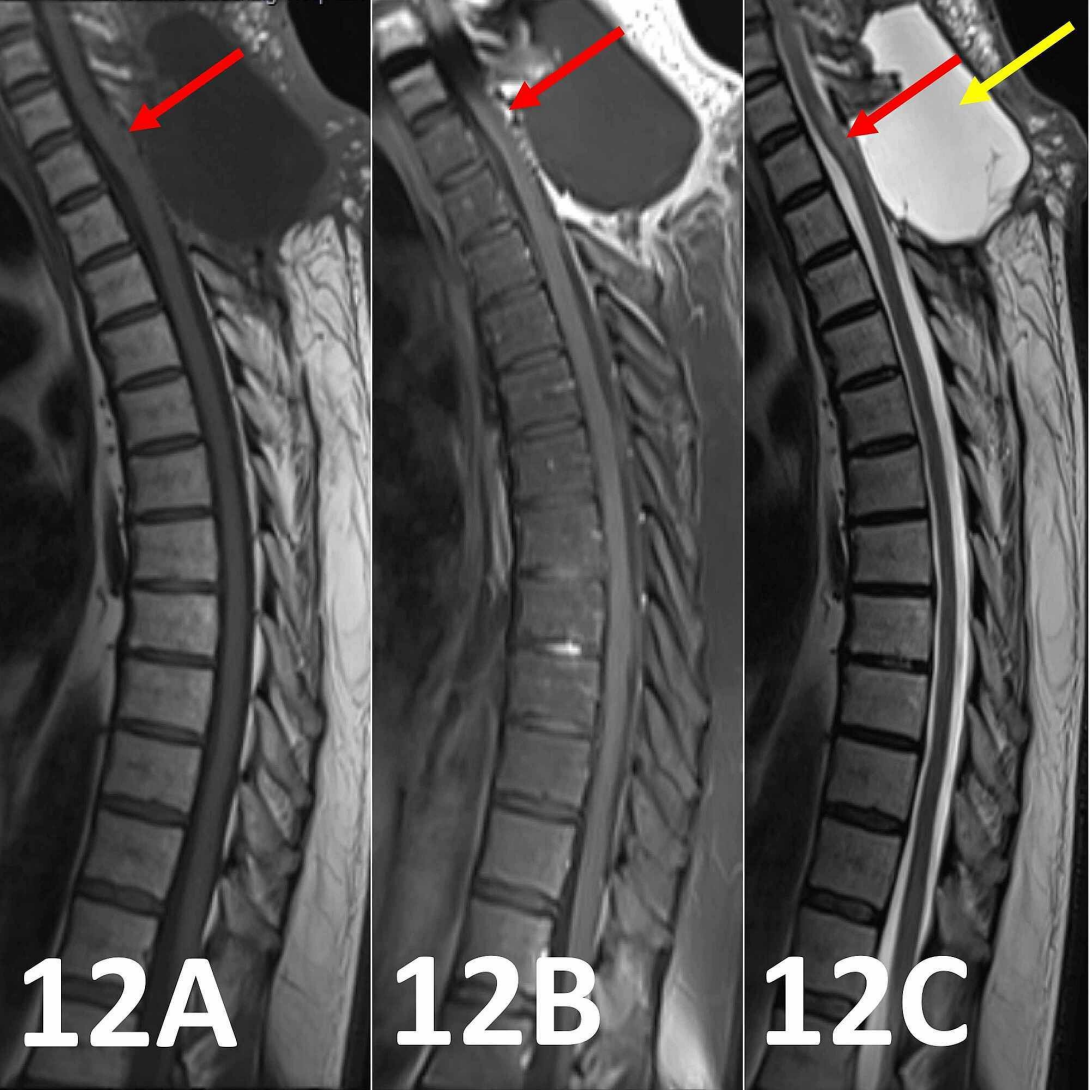
MRI of the thoracic spine with contrast 10 days post-operatively. (12A and 12B) Pre- and post-contrast sagittal sequences demonstrate equivocal punctate dots of remnant enhancement within the spinal cord at the resection site indicating near-complete resection of the enhancing tumoral component (red arrows). (12C) High T2 (and low T1 in Figures 12A, 12B) contained collection consistent with a post-operative pseudomeningocele abutting the posterior dura with few intrinsic septations (yellow arrow). The spinal cord is tethered to the posterior dura at the T1 vertebral level. Minor high T2 intrinsic spinal cord signal from the inferior endplate of C7 to the superior endplate of T2 has improved in comparison to preoperative MRI (red arrow).

Early (10 days) post-operative MRI imaging revealed near-complete resection of the multiple spinal schwannomas (Figures 12A-12C).

Six months post-operatively, the pain resolved completely. Spinal MRI imaging showed no tumor residuum or recurrence.

## Discussion

Spinal schwannomas (less commonly called neurinomas or neurilemmomas) are benign encapsulated nerve sheath neoplasms that arise from Schwann cells of macroscopically recognizable nerve fibers. These lesions exhibit an eccentric growth pattern, with the nerve fiber itself usually being incorporated into the tumor capsule. These lesions are considered the most common tumors of peripheral nerves, accounting for nearly one-third of all spinal neoplasm [1,2]. The vast majority of schwannomas (90-95%) remain solitary and sporadic, whereas a rare but characteristic association with NF2 and schwannomatosis has been described [1,3-5]. The peak presentation occurs in the fifth to sixth decades of life, with the condition expressing no sex predilection. However, when schwannomas are associated with NF2, they usually present as early as the third decade of life.

Characteristically, schwannomas are intradural extramedullary WHO grade I spinal tumors composed entirely of well-differentiated eosinophilic Schwann cells. Intradural intramedullary schwannomas, as in our case, are extremely rare and represent only 0.3%-1% of all the spinal schwannoma population. The characteristic lack of an intramedullary component has been historically attributed to the fact that Schwann cells do not routinely exist within the spinal cord [6,7]. Unlike their extramedullary counterparts, intramedullary schwannomas exhibit a 3:1 male predilection and present relatively earlier in the fourth decade of life [2,3]. Given the lack of Schwann cells in the normal spinal cord, various theories have been postulated to explain the occurrence of such intramedullary spinal component. These theories include the central inclusion of Schwann cells during embryological development, subpial Schwann cell extension along the perivascular nerve plexus, Schwann cells differentiation from mesenchymal CNS elements, and proliferation of Schwann cells after trauma or inflammatory disease. The validity of these theories remains controversial, and further research in this field is required to explain the development of such intramedullary extension [6,8,9].

Contrast-enhanced MRI is the most sensitive and specific imaging modality to evaluate possible spinal column lesions. Classically, imaging reveals a well-circumscribed T1 isointense (75%) or T1 hypointense (25%) signal characteristic and T2 hyperintense nodular intradural extramedullary lesions with associated intense contrast enhancement and a characteristic dumbbell morphology when neuroforaminal extension is present [10-12]. When in contact with an osseous margin, benign bone erosional effects and remodeling may be observed [10,13,14]. In most instances, spinal schwannomas are radiologically non-distinguishable from neurofibromas, but the presence of hemorrhage, intrinsic vascular changes (e.g. thrombosis and sinusoidal dilatation), cyst formation, and fatty degeneration favors schwannomas. Once a spinal tumor is identified, MRI of the head and the remainder of the spine is routinely obtained to identify possible additional neoplasms that may be found in patients with neurofibromatosis (e.g., meningiomas and ependymomas) and exclude brain tumors that may spread to lower levels of the spinal cord (i.e., drop metastasis).

The possession of an intramedullary component makes differentiating extramedullary spinal schwannomas with intramedullary extension, as in our case, from other intramedullary spinal neoplasms a radiological challenge. Common intramedullary tumors such as astrocytomas and ependymomas expand the spinal cord and often have an associated polar cyst, which makes them radiologically distinguishable, but if these lesions express an exophytic component, they may not be easily be differentiated from intramedullary schwannomas. A predominantly extramedullary component or a thickened enhancing nerve root lesion should always increase the suspicion for schwannomas [15,16]. In the literature, the presence of a nerve root component is considered a highly specific but uncommon finding that supports the diagnosis of schwannoma and may be responsible for the root pain, leading to the clinical presentation, as in the presented case [6].

Although MRI can define the anatomic interface between the tumor and spinal cord, it cannot reliably establish the histopathological entity of the neoplasm [1]. Histologically, schwannomas are benign encapsulated neoplasms. In contradistinction to neurofibromas, which arise from within the nerve, schwannomas arise eccentrically from their parent nerve splaying and displacing the nerve fibers along their surface. Microscopically, these lesions are composed of spindle cells arranged in short, intersecting fascicles. Nuclear palisading is a typical feature and, when pronounced, results in the formation of Verocay bodies. Antoni A and B are two histopathological architectures that have been described and usually co-exist [17,18]. The Antoni A regions consist of compact areas of spindle cells with pink cytoplasm. These alternate with looser Antoni B tissue, comprises cells showing clear, vacuolated cytoplasm due to lipid accumulation. Myxoid change is not prominent, except in myxoid variants [1,3,19].

The natural history of schwannomas is of a slowly growing lesion. Although surgery is the treatment of choice when lesions are large and symptomatic, an initial conservative approach can be adopted for smaller, incidentally detected neoplasms. As schwannomas do not infiltrate the parent nerve, intra-operative dissection from the parent nerve is usually feasible. Post-operatively, one in five patients remains symptom-free at follow-up, whereas 80% of patients display varying degrees of neurologic compromise such as local/radicular pain and paraparesis [20]. Recurrence of schwannomas is unusual. Schwannomas rarely undergo malignant transformation or negatively influence patient life expectancy in comparison to the general population.

## Conclusions

Intradural intramedullary schwannomas are extremely rare and represent <1% of all the spinal schwannoma population. These neoplasms may be either sporadic or associated with schwannomatosis or NF2. Schwannomas are typically slow-growing tumors whose clinical manifestations depend upon the level of the spinal cord involved. Symptoms can progress rapidly if spinal cord compression occurs. Contrast-enhanced MRI is the most sensitive and specific imaging modality to evaluate possible spinal column lesions. Surgical resection is the treatment of choice for most patients. Post-operatively, around 20% of patients remain symptom-free at follow-up, whereas 80% display varying degrees of neurologic symptoms.
